# Field-induced spin-density wave beyond hidden order in URu_2_Si_2_

**DOI:** 10.1038/ncomms13075

**Published:** 2016-10-20

**Authors:** W. Knafo, F. Duc, F. Bourdarot, K. Kuwahara, H. Nojiri, D. Aoki, J. Billette, P. Frings, X. Tonon, E. Lelièvre-Berna, J. Flouquet, L.-P. Regnault

**Affiliations:** 1 Laboratoire National des Champs Magnétiques Intenses, UPR 3228, CNRS-UPS-INSA-UGA, 143 Avenue de Rangueil, 31400 Toulouse, France; 2 Service de Modélisation et d'Exploration des Matériaux, Université Grenoble Alpes et Commissariat á l'Energie Atomique, INAC, 17 rue des Martyrs, 38054 Grenoble, France; 3 Institute of Quantum Beam Science, Ibaraki University, Mito 310-8512, Japan; 4 Institute for Materials Research, Tohoku University, Sendai 980-8578, Japan; 5 Institute for Materials Research, Tohoku University, Ibaraki 311-1313, Japan; 6 Service Photonique, Electronique et Ingénierie Quantiques, Université Grenoble Alpes et Commissariat à l'Energie Atomique, INAC, 17 rue des Martyrs, 38054 Grenoble, France; 7 Institut Laue-Langevin, 71 Avenue des Martyrs, CS 20156, 38042 Grenoble, France

## Abstract

URu_2_Si_2_ is one of the most enigmatic strongly correlated electron systems and offers a fertile testing ground for new concepts in condensed matter science. In spite of >30 years of intense research, no consensus on the order parameter of its low-temperature hidden-order phase exists. A strong magnetic field transforms the hidden order into magnetically ordered phases, whose order parameter has also been defying experimental observation. Here, thanks to neutron diffraction under pulsed magnetic fields up to 40 T, we identify the field-induced phases of URu_2_Si_2_ as a spin-density-wave state. The transition to the spin-density wave represents a unique touchstone for understanding the hidden-order phase. An intimate relationship between this magnetic structure, the magnetic fluctuations and the Fermi surface is emphasized, calling for dedicated band-structure calculations.

## Abstract (web summary)

The strongly-correlated electron system URu_2_Si_2_ possesses a hidden-order phase whose order parameter remains unidentified. Here, the authors demonstrate the development of spin-density-wave phases in URu_2_Si_2_ under high magnetic fields, providing a potential in-road to understanding this system.

For since three decades, a huge experimental and theoretical effort has been employed to try to determine the ground state of the heavy-fermion material URu_2_Si_2_, where a second-order phase transition occurs at the temperature *T*_0_=17.5 K and for which the order parameter has resisted experimental identification^1^. The case of URu_2_Si_2_—which is also superconducting^2^—is not isolated, since mysterious electronic phases, identified as ‘pseudo-gap' and ‘nematic' phases, have also been reported in high-temperature cuprate and iron-based superconducting materials^3^,^4^. Initially, a small magnetic moment of 0.02 Bohr magneton per uranium (*μ*_B_/U) antiferromagnetically aligned along **c** and modulated with the wavevector **k**_0_=(0 0 1) has been considered as the order parameter of URu_2_Si_2_ below *T*_0_ (ref. 5). However, this moment is too small to account for the large entropy associated with the transition and is now suspected to result from sample inhomogeneities^6^. In the hidden-order phase, the low-energy magnetic fluctuations are peaked at the hot wavevectors **k**_0_ and **k**_1_=(0.6 0 0)^7^,^8^, and the Fermi surface presents nesting with these two wavevectors^9^,^10^,^11^,^12^,^13^,^14^,^15^, suggesting an itinerant origin of the magnetic properties. Recent experimental works have been interpreted as the signature of nematicity, that is, a correlated electronic state associated with a breaking of the fourfold symmetry, in the hidden-order phase^16^,^17^,^18^.

The application of extreme conditions permits to modify the ground state of a system and get new insights about it. In URu_2_Si_2_ under pressure, the hidden-order state is replaced by an antiferromagnetic phase^19^, whose order parameter is a moment of ≃0.3–0.4 *μ*_B_/U aligned antiferromagnetically along **c** with the wavevector **k**_0_ (refs 20, 21). From the similar Fermi surfaces observed in the hidden-order and pressure-driven antiferromagnetic phases, it has been proposed that the hidden order, although of unknown nature, has a periodicity of wavevector **k**_0_ (ref. 19). Under a high magnetic field applied along the easy magnetic axis **c**, a cascade of first-order phase transitions at the fields *μ*_0_*H*_1_=35 T, *μ*_0_*H*_2_=36/37 T (rising/falling fields), and *μ*_0_*H*_3_=39 T, leads to a polarized paramagnetic regime above *H*_3_, where a magnetization of ≈1.4 *μ*_*B*_/U is reached (Fig. 1b)^22^,^23^. Quantum oscillations probes have shown that a magnetic field along **c** induces Fermi surface instabilities deep inside the hidden-order phase, as well as at the magnetic transitions *H*_1_, *H*_2_ and *H*_3_ (refs 24, 25, 26, 27, 28). By comparison with 3*d* itinerant magnets, a novelty in heavy-fermion compounds is that a large magnetic polarization can be induced by a magnetic field, opening the path to new phases driven by combined changes of magnetic order and Fermi surface. Such interplay of metamagnetic and Lifshitz transitions has been evidenced in the heavy-fermion paramagnet CeRu_2_Si_2_ (ref. 29).

In Rh-doped U(Ru_0.96_Rh_0.04_)_2_Si_2_, no hidden-order phase is reported, but first-order transitions are also induced at magnetic fields of 26 and 37 T applied along **c** (refs 30, 31, 32). Between 26 and 37 T, a squared up–up–down ferrimagnetic phase was deduced from Bragg peaks (harmonics) at the wavevector **k**_2_=(2/3 0 0) and at the Brillouin zone centre **k**_ZC_=(0 0 0)^33^. However, the extrapolation to the pure case of URu_2_Si_2_ is not straightforward, as doping may modify the low-carrier content of the pure lattice, and, thus, the Fermi surface topology, leading to different nesting effects. Here thanks to a new cryomagnet allowing neutron diffraction up to 40 T, we have determined the magnetic structure of URu_2_Si_2_ in fields between 35 and 39 T. A tilt by 4.2° of the field direction from **c** in the (**c**,**a**) plane allowed accessing a large number of momentum transfers **Q**, with almost no modification of the magnetic phase diagram expected for **H** || **c** (refs 26, 34). The relationship between the high-field magnetic structure, the low-energy magnetic fluctuations and the Fermi surface is emphasized, appealing for new band-structure calculations describing the electronic properties of URu_2_Si_2_.

## Results

### Spin-density wave with wavevector k_1_

**Figure 1:**
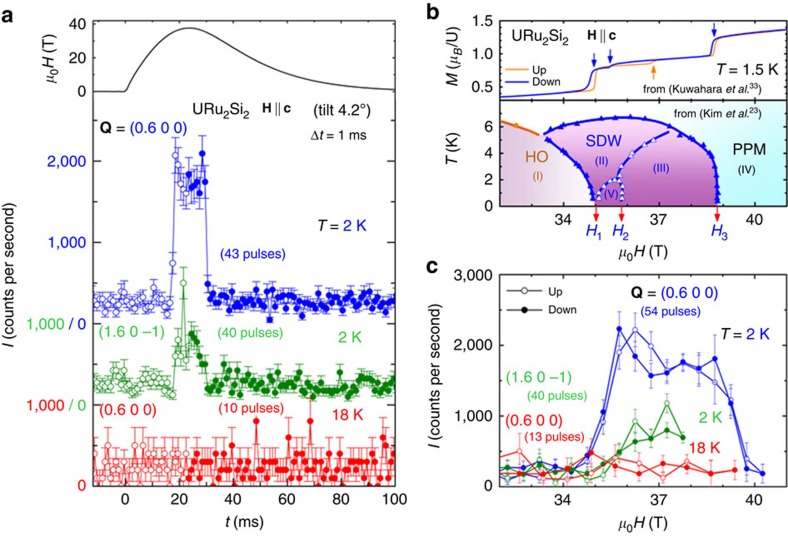
Signatures of the field-induced magnetic phases. (**a**) Time profile of a field pulsed up to 38 T, and time dependence of the diffracted intensity at **Q**=(0.6 0 0) and *T*=2 K, **Q**=(1.6 0 −1) and *T*=2 K, and **Q**=(0.6 0 0) and *T*=18 K, in fields up to 38 T, integrated within time steps Δ*t*=1 ms. The error bars Δ*I* are given by the square root of the neutron counts (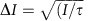, where τ is the total accumulation time over several pulses). (**b**) Magnetization versus magnetic field at *T*=1.5 K (from ref. 33) and magnetic field–temperature phase diagram deduced from magnetoresistivity versus temperature and versus field, in falling fields (from ref. 23). (**c**) Field dependence of the same diffracted intensities in fields up to 40.5 T, integrated within field steps Δ(*μ*_0_*H*)=0.5 T. Open and full symbols correspond to rising and falling fields, respectively. The magnetic field has been applied along the easy magnetic axis **c** with a tilt of 4.2° along **a**.

**Figure 2:**
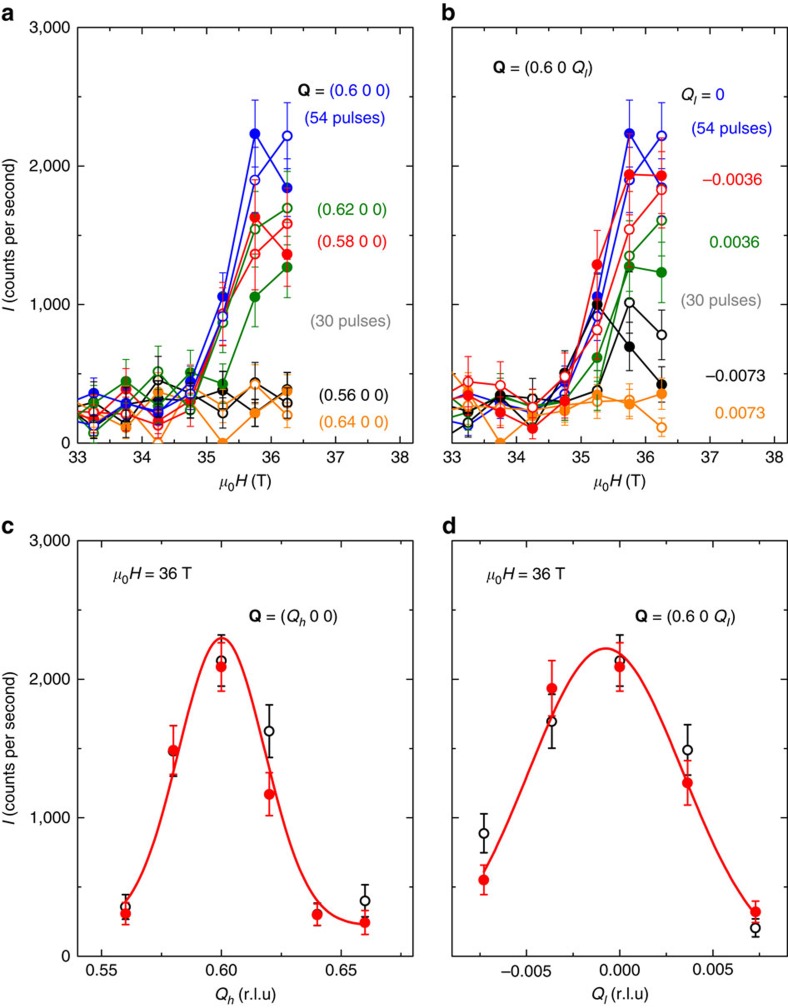
High-field neutron diffraction at momentum transfers close to Q_1_, at *T*=2 K. Magnetic field dependence of the neutron-diffracted intensity (**a**) at momentum transfers **Q**=(*Q*_*h*_ 0 0) and (**b**) at momentum transfers **Q**=(0.6 0 *Q*_*l*_), in fields up to 36.2 T, within field steps Δ(*μ*_0_*H*) of 0.5 T. (**c**) *Q*_*h*_ and (**d**)*Q*_*l*_ scans around the momentum transfer **Q**_1_=(0.6 0 0) under a magnetic field *μ*_0_*H*=36 T (field window Δ(*μ*_0_*H*)=1 T). The error bars Δ*I* are given by the square root of the neutron counts (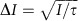). Open and full symbols correspond to rising and falling fields, respectively. The magnetic field **H** has been applied along the easy magnetic axis **c** with a tilt of 4.2° along **a**.

Figure 1a shows the time profile of a magnetic field pulsed up to 38 T, as well as the time dependence of the diffracted neutron intensities recorded during 38-T pulses at the temperatures *T*=2 and 18 K and at **Q**=(0.6 0 0) and (1.6 0 −1). These momentum transfers are satellites of wavevector **k**_1_=(0.6 0 0)=**Q**–**τ** around the structural Bragg positions **τ**=(0 0 0) and (1 0 −1), respectively. The high-field enhancement of the diffracted intensity at *T*=2 K, absent at *T*=18 K, shows that long-range ordering with wavevector **k**_1_=(0.6 0 0) is established at high field and low temperature. Figure 1c emphasizes in the window 32–41 T the field dependence of the diffracted neutron intensities measured up to 40.5 T. At *T*=2 K, the diffracted intensity with wavevector **k**_1_ is enhanced in fields higher than *μ*_0_*H*_1_=35 T and drops down to the background level in fields higher than *μ*_0_*H*_3_=39 T. No hysteresis is observed at *H*_1_ and *H*_3_ within the neutron counting statistics, and a slight field-induced decrease of intensity, suggesting a modification of magnetic ordering, is visible at *μ*_0_*H*_2_≈36 T for rising field and at *μ*_0_*H*_2_≈37 T for falling field, in agreement with the hysteresis observed by magnetization^33^ (Fig. 1b). Figure 2 presents the high-field neutron-diffracted intensity at momentum transfers **Q** in the vicinity of (0.6 0 0) at *T*=2 K. The field dependence of the intensity at momentum transfers (*Q*_*h*_ 0 0) and (0.6 0 *Q*_*l*_) is shown in fields up to 36.2 T in Fig. 2a,b, respectively. Figure 2c,d presents *Q*_*h*_—and *Q*_*l*_—scans at *μ*_0_*H*=36 T, confirming that the field-induced magnetic intensity is peaked at **Q**=(0.6 0 0). From the intensity of the magnetic Bragg peaks induced at **Q**=(0.6 0 0) and **Q**=(1.6 0 −1) at *μ*_0_*H*=36 T>*μ*_0_*H*_1_, we extract the amplitude 2*M*(**k**_1_)≃0.5±0.05 *μ*_*B*_/U of the sine modulation with wavevector **k**_1_ of the magnetic moment *M(***k**_1_,**r**)=2*M*(**k**_1_)cos(2π**k**_1_·**r**+*ϕ*_1_), where **r** is an U-ion position and *ϕ*_1_ the associated phase (cf. Supplementary Note 1). This result is in agreement with the high-field magnetization^22^, since the amplitude 2*M*(**k**_1_) extracted here at 36 T is of same order than the variation of magnetization Δ*M*≃0.4 *μ*_*B*_/U (estimated from 36–39 T) induced by the quench of the field-induced ordered moments into a polarized regime above *μ*_0_*H*_3_=39 T.

**Figure 3:**
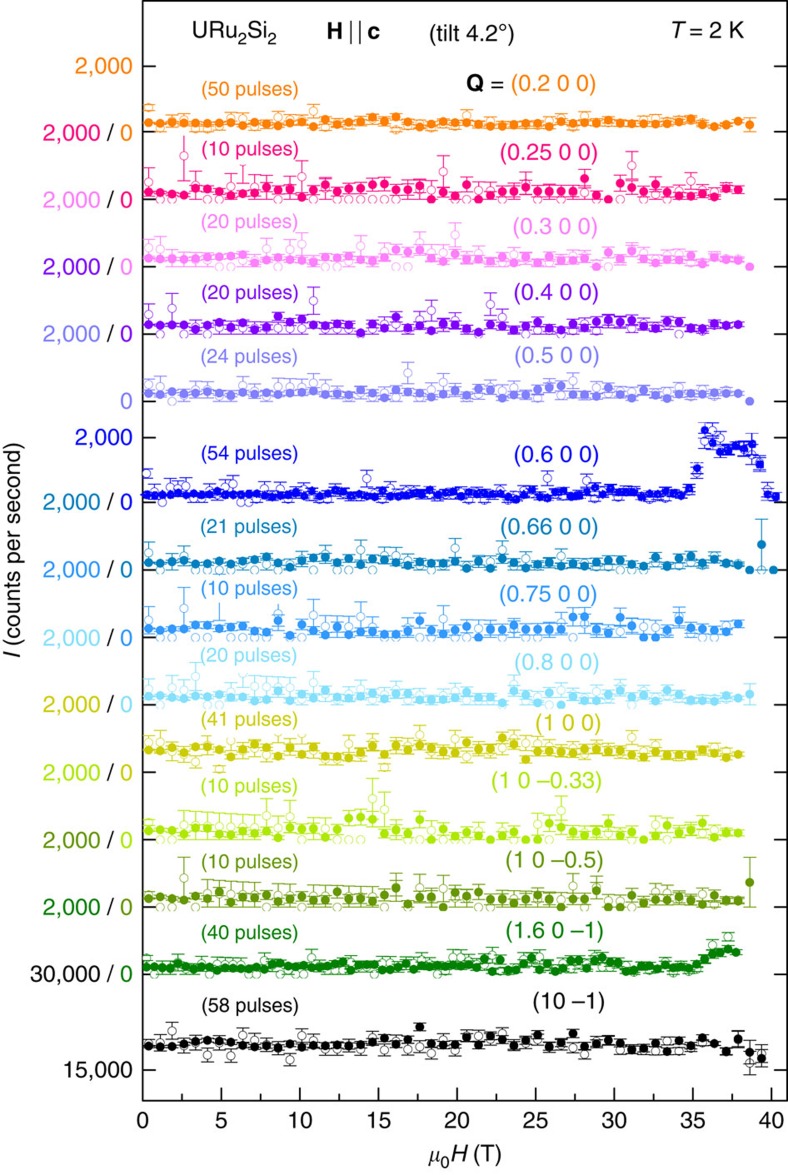
Neutron-diffracted intensity for a large set of momentum transfers. Magnetic fields were applied up to 40.5 T, at a temperature *T*=2 K. The error bars Δ*I* are given by the square root of the neutron counts (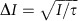). Open and full symbols correspond to rising and falling fields, respectively. The magnetic field **H** has been applied along the easy magnetic axis **c** with a tilt of 4.2° along **a**.

The field dependence of the diffracted intensity is presented in Fig. 3 for a large set of momentum transfers **Q** from (*Q*_*h*_ 0 0), (*Q*_*h*_ 0 −1) and (1 0 *Q*_*l*_) scans in the reciprocal space at *T*=2 K. Except at **Q**=(0.6 0 0) and (1.6 0 −1) corresponding to **k**_1_, no field-induced change of the neutron-diffracted intensity is observed at the different probed momentum transfers. In particular, no change of intensity occurs in high field at the harmonics *n***k**_1_, where *n*≠1 is an integer, including the Brillouin zone centre **k**_ZC_=(0 0 0), within an error of ∼5–10% of the induced intensity at **k**_1_ (cf. Supplementary Table 1). The absence of harmonics supports that the high-field magnetic structure is a spin-density wave, that is, a sine-modulated magnetic structure, in opposition with a squared-modulated structure, where harmonics of the main wavevector would lead to full moments, either up or down, on the U sites (cf. Supplementary Note 2). Spin-density-wave modulations of the magnetic moment are generally observed at low temperature in itinerant magnets at zero field, as chromium^35^, or, in a magnetic field, as the heavy-fermion superconductor CeCoIn_5_ (ref. 36) and the low-dimensional magnet Sr_3_Ru_2_O_7_ (ref. 37). However, in the present case, we cannot fully exclude the possibility of a ‘special' sequence of squared Ising moments, for which high-order harmonics *n***k**_1_ would all have intensities smaller than our error bar, that is, 5–10% of the magnetic intensity at **k**_1_. The absence of neutron Bragg peaks at **k**_2_=(2/3 0 0) and **k**_ZC_ is in contradiction with the up–up–down squared modulation proposed from nuclear magnetic resonance (NMR) measured on URu_2_Si_2_ in the field window 35.5–36.5 T (ref. 38). The NMR spectrum, composed of a central narrow peak and two broad satellites, is not compatible with a single-**k** spin-density wave, which would lead either to a few discrete and thin peaks (if **k**_1_ is commensurate) or to a double-horn line shape (if **k**_1_ is incommensurate)^38^. However, an incommensurability of **k**_1_ (that is, a slight deviation from (0.6 0 0) compatible with our neutron resolution Δ**k**≃(±0.01±0.05±0.003)), combined with a multi-**k** character of the spin-density wave, could induce peculiar features, as a central peak, in the NMR spectrum^39^, and could possibly reconcile the NMR and neutron studies. Such multi-**k** structure would imply a conservation of the fourfold symmetry, that is, of the equivalence between *x* and *y*, in the high-field spin-density-wave phase, in contrast with the twofold symmetry proposed in refs 16, 17 for the low-field hidden-order phase.

### Absence of a ferromagnetic Bragg peak

Surprisingly, the neutron-diffracted intensity at the Brillouin zone centre **k**_ZC_ is field invariant, while the magnetization *M* is strongly enhanced above 35 T (ref. 22). Usually, a magnetic Bragg peak develops at **k**_ZC_ and has an intensity ∝*M*^2^. From *M*≃0.5–1 *μ*_*B*_/U in the field window 35–39 T, and assuming a magnetic form factor corresponding to localized *f* electrons (estimated from a low-field study of the magnetic form factor^40^), one expects a magnetic intensity of ≃2,000–8,000 counts per s, in addition to the non-magnetic Bragg peak of ≃19,000 counts per s, at **Q**=(1 0 −1) corresponding to **k**_ZC_ (cf. Supplementary Note 1). However, no such change of intensity is observed here at **Q**=(1 0 −1), within an error of 200 counts per s. Several scenarios could explain this unexpected feature. (i) The absence of a magnetic Bragg peak at **k**_ZC_ could result from a strongly-reduced magnetic form factor *f*_*M*_ related to itinerant electrons. Since the observation of magnetic Bragg peaks at **Q**=(0.6 0 0) and (1.6 0 −1) demonstrates that nearly localized electrons with *f*_*M*_≲1 drive the high-field spin-density wave of wavevector **k**_1_, scenario (i) would imply that the high-field magnetism would be localized, or nearly localized, at **k**_1_ but itinerant at **k**_ZC_. (ii) Alternatively, the absence of magnetic Bragg peak at **k**_ZC_ could be explained by field-induced magnetic excitations at **k**_ZC_, which would contribute to the enhanced magnetization (cf. Kramers–Kronig relationship^41^), but not to the magnetic Bragg peak at **k**_ZC_. (iii) Finally, this absence could also result from a high-field decrease of the nuclear Bragg peak at **Q**=(1 0 −1) (related via local stress effects to a change of some atomic positions), which would counteract the increase of magnetic intensity at **k**_ZC_. The missing magnetic Bragg intensity at **k**_ZC_ constitutes an open question and will merit further studies.

### Relationship with Fermi surface nesting

In an itinerant picture of magnetism, long-range magnetic ordering with a wavevector **k**_*m*_ can be related to a partial or complete nesting of two parts of the Fermi surface. The dual localized itinerant behaviour is a central issue in heavy-fermion metals, where the magnetic properties often result from itinerant *f* electrons. In URu_2_Si_2_, 5 *f* electrons hybridize with electrons from the conduction bands and modifications of the Fermi surface and carrier mobility accompany the establishment of the hidden-order phase at the temperature *T*_0_ (refs 26, 42, 43, 44, 45). The itinerancy of the *f* electrons has already been shown to be a key ingredient for the magnetic properties of this system. Angle-resolved photoemission spectroscopy and band-structure calculations showed that Fermi surfaces centred on the Г and Z points are nested with the wavevector **k**_0_ (refs 9, 10, 11, 12, 15). In refs 9, 15, a nesting with the wavevector **k**_1_ between Fermi surface ‘petals' around the Г point has been identified. In refs 11, 12), a nesting with the wavevector (0.4 0 0) between these petals and a Fermi surface centred on the Г point has also been found. In ref. 14, another picture has been proposed, with a nesting with wavevector **k**_0_ between small Fermi pockets centred on the X points (defined in the first Brillouin zone) and a nesting with wavevector **k**_1_ between Fermi surfaces centred on the Г and Z points. Despite these nesting of the Fermi surface, no long-range magnetic order has been reported yet in URu_2_Si_2_ in its hidden-order phase. Instead of long-range magnetic order, inelastic magnetic excitations at the wavevectors **k**_0_ and **k**_1_ have been observed by neutron scattering in the hidden-order phase^5^,^6^,^7^,^8^. Their relationship with nesting effects and long-range magnetic ordering will be discussed below.

Assuming a high-rank multipolar order with wavevector **k**_0_, which cannot be seen directly by neutron diffraction, band-structure calculations resulted in a Fermi surface of the hidden-order state, which is very similar to that of the pressure-induced antiferromagnetic state^9^, in accordance with the experimental observations^19^. Under a high magnetic field applied along **c**, successive Fermi surface modifications occur inside the hidden-order phase and at the transition fields *H*_1_, *H*_2_ and *H*_3_ delimiting the high-field phases^24^,^25^,^26^,^27^,^28^. Several Fermi surface instabilities in fields smaller than *μ*_0_*H*_1_=35 T, that is, inside the hidden-order phase^24^,^26^,^27^,^28^, are due to Lifshitz transitions and suggest a progressive modification of Fermi surface nestings. Resulting from the Fermi surface reconstruction at *H*_1_, the Fermi surface nesting with **k**_1_ is probably reinforced, leading to the onset of the spin-density wave observed here with the wavevector **k**_1_.

### Magnetic fluctuations as a precursor of magnetic ordering

**Figure 4:**
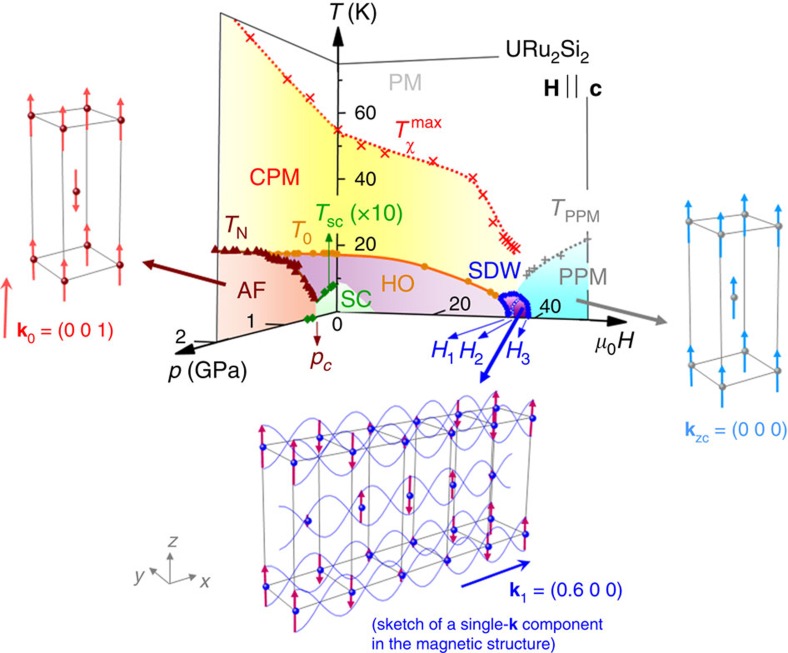
Temperature–magnetic field and temperature–pressure phase diagrams of URu_2_Si_2_. PM, CPM and PPM are the high-temperature, the low-temperature correlated and the high-field polarized paramagnetic regimes, respectively, HO and SC the hidden-order and superconducting phases, respectively, AF the antiferromagnetic state, and SDW the spin-density-wave state. For the AF, SDW and PPM regimes, a sketch of the magnetic structure is shown, where the spheres correspond to the U ions and the arrows represent their magnetic moments. For clarity, a single-component (wavevector **k**_1_=(0.6 0 0)) sine modulation is shown for the SDW, which is suspected to be multi-**k**. Data points in the phase diagram have been compiled from refs 19, 22, 23, 31, 50.

Figure 4 presents the magnetic phase diagram of URu_2_Si_2_ under pressure and magnetic field, over a wide range of temperatures up to 75 K. At zero field and ambient pressure, URu_2_Si_2_ is in a correlated paramagnetic regime below *T*_*χ*_^max^=55 K at which the static magnetic susceptibility *χ* is maximal^22^. The correlations are accompanied by a strong and broad magnetic-fluctuation spectrum peaked at the wavevector **k**_1_, which progressively sets at temperatures of the order of *T*_*χ*_^max^ (ref. 8). When the system enters in its hidden-order phase, the magnetic fluctuations give rise to sharp inelastic spectra at the wavevectors **k**_0_ and **k**_1_, with the gaps Δ*E*_0_≃1.7 meV and Δ*E*_1_≃4.5 meV, respectively, at low temperature. Below *T*_0_, the intensity of the magnetic fluctuations at **k**_0_ suddenly develops, while the intensity of the fluctuations at **k**_1_, as indicated by the static susceptibility *χ′*(**k**_1_, *E*=0), drops by 20% (refs 8, 46).

The magnetic fluctuations peaked at the hot wavevectors **k**_0_ and **k**_1_ indicate the proximity of quantum-phase transitions associated with long-range ordering with the same wavevectors. Such precursory role of the magnetic fluctuations has been emphasized in the heavy-fermion paramagnet CeRu_2_Si_2_, where doping (La, Rh or Ge) and magnetic field permit to tune competing quantum instabilities (cf. ref. 47 and refs therein). With pressure^20^,^21^ (as well as Rh doping^30^), URu_2_Si_2_ can be tuned to antiferromagnetic long-range order with wavevector **k**_0_. The disappearance of the hidden order in URu_2_Si_2_ under pressure coincides with a transfer of weight from magnetic fluctuations with wavevector **k**_0_ into antiferromagnetic order with the same wavevector^48^,^49^. Contrary to those with **k**_0_, the fluctuations with wavevector **k**_1_ survive under pressure^48^ and their progressive variation may be in relationship with the continuous evolution of *T*_*χ*_^max^ (ref. 50). With magnetic field, a spin-density wave is stabilized with the wavevector **k**_1_. Both *T*_*χ*_^max^ and *T*_0_ vanish in the quantum critical area *μ*_0_*H*≃35–39 T (ref. 22) where long-range magnetic ordering sets in. In the polarized regime above *μ*_0_*H*_3_=39 T, from the magnetization >1.4 *μ*_*B*_/U, one can expect a loss of intersite magnetic correlations at **k**_0_ and **k**_1_, the magnetic excitations becoming wavevector-independent, that is, of local nature. This picture is compatible with the observation in fields up to 17 T that the gap in the excitations at **k**_0_ increases with *H*, while that in the excitations at **k**_1_ remains constant, their extrapolations converging at ≃35 T (ref. 51). In the critical field window (35 and 39 T), the enhancement of the Sommerfeld coefficient^22^ is the signature of critical fluctuations, presumably of magnetic origin (cf. CeRu_2_Si_2_, where critical magnetic fluctuations were reported at the La-doping- and magnetic-field-induced instabilities^52^,^53^). However, inelastic neutron scattering under steady fields up to 40 T, which is far beyond the present state of the art (26 T achieved recently at the neutron source in Berlin^54^), would be needed to unveil the high-field evolution of the magnetic fluctuations in URu_2_Si_2_.

## Discussion

We succeeded in detecting a high-magnetic-field switch from hidden-order to spin-density-wave phases in URu_2_Si_2_. This result illustrates the dual localized and itinerant facets of the 5*f* electrons in this system. The relationship between Fermi surface nesting and the magnetic wavevector **k**_1_ of the spin-density wave, as well as the cascade of Lifshitz transitions induced by the high-field magnetic polarization^24^,^25^,^26^,^27^,^28^, show the itinerant character of the 5*f* electrons. The neutron-diffracted intensity with the wavevector **k**_1_ is compatible with the magnetic form factor *f*_*M*_ corresponding to U^3+^ or U^4+^ configurations^40^ and highlights the localized character of the 5*f* electrons. Knowing that a change of the Fermi surface in the polarized regime of URu_2_Si_2_ was detected indirectly by Hall effect^55^ and directly by quantum oscillations experiments^25^, a challenge is to determine whether the missing magnetic Bragg peak at the wavevector **k**_ZC_=(0 0 0) in a high magnetic field could be related to a change in the itinerancy of the *f*-electrons.

A relationship between the long-range magnetic order, the Fermi surface and the magnetic fluctuations has been highlighted. In the hidden-order phase, one may suspect ‘inelastic' nestings between electronic band surfaces of different energies, to be connected with the observed gapped magnetic excitations at the wavevectors **k**_0_ and **k**_1_ (refs 5, 6, 7, 8). These excitations are precursors of the long-range ordered states induced under magnetic field and pressure. Under a magnetic field, the transformation of the paramagnetic state with gapped magnetic excitations into a spin-density wave with wavevector **k**_1_ might be related to the stabilization of Fermi surface nesting with the wavevector **k**_1_. Under pressure, a modification of the Fermi surface nesting with the wavevector **k**_0_ might also lead to long-range magnetic order with the wavevector **k**_0_. Additional effects, as modifications of the crystal field, could also play a role in the stabilization of magnetically ordered phases.

Interestingly, the effect of Rh doping leads to a decrease of *T*_0_ and *T*_*χ*_^max^ (refs 30, 31) similar to the magnetic field effect, and to antiferromagnetic order with wavevector **k**_0_ (for Rh contents 0.02≤*x*≤0.03) similar to the pressure effect. The main wavevector **k**_2_=(2/3 0 0) of the high-field squared magnetic structure of U(Ru_0.96_Rh_0.04_)_2_Si_2_ (ref. 33) is rather close to the wavevector **k**_1_=(0.6 0 0) of the spin-density wave of URu_2_Si_2_, confirming a relationship between these two magnetic phases. The differences between these wavevectors might result from subtle differences in the band structures of the two compounds, due to the doping sensibility of their low-carrier density.

Our observation of a spin-density wave in magnetic fields between 35 and 39 T will certainly push to develop models incorporating on equal basis the Fermi surface topology and the magnetic interactions. With the objective to describe competing quantum-phase transitions between the hidden-order and long-range ordered phases, such models will be a basis to solve the hidden-order problem. From an instrumental point of view, this work paves the way for neutron magnetic diffraction on weak-moment systems under pulsed magnetic field up to 40 T.

## Methods

### Samples

The single crystal of mass *m*≃1 g and dimensions 6 × 4 × 4 mm^3^ used in this study has been grown by the Czochralski technique in a tetra-arc furnace. It has been characterized by X-ray Laue. A sample from the same batch was checked by electrical resistivity, showing a residual resistivity ratio of ∼50 and a superconducting temperature *T*_c_ of ∼1.5 K, indicating its high quality. A slit perpendicular to the basal plane was cut into the sample to reduce the surface exposed to the pulsed field and, thus, self-heating of the sample by eddy currents.

### 40-T cryomagnet

A new long-duration (rising time of 23 ms and total time of >100 ms) and high duty cycle (40, 36 and 31-T shots can be repeated every 10, 7 and 5 min, respectively) 40-T conical pulsed magnet developed by the LNCMI-Toulouse has been used. A cryostat specially designed by the ILL-Grenoble for this magnet allowed reaching temperatures down to 2 K, and the diffraction experiment was carried out on the triple-axis spectrometer IN22 (Collaborative Research Group-CEA at the ILL, Grenoble) operated in a double-axis mode with an incident neutron wavelength of 1.2 Å. At each momentum transfer, the field dependence of the neutron intensities has been extracted by summing the data accumulated over a few tens of pulsed-field shots, with either constant time- or constant field-integration windows (the rise and fall of the pulsed field were analysed separately).

### Data availability

The data that support the findings of this study are available from the corresponding author on request.

## Additional information

**How to cite this article**: Knafo, W. *et al*. Field-induced spin-density wave beyond hidden order in URu_2_Si_2_. *Nat. Commun.* **7**, 13075 doi: 10.1038/ncomms13075 (2016).

## Supplementary Material

Supplementary Information Supplementary Table 1, Supplementary Note 1-2 and Supplementary References
