# Supplementary material for: Field-induced spin-density wave beyond hidden order in URu2Si2
Source: Nat Commun. 2016 Oct 20;7:13075. doi: 10.1038/ncomms13075 (PMC5080431; doi:10.1038/ncomms13075)
Supplement: Supplementary Information — Supplementary Table 1, Supplementary Note 1-2 and Supplementary References [file ncomms13075-s1.pdf]

Supplementary Table 1. Neutron diffracted intensities I_0 and I_m summed over the field windows [0;34T] and [35;39T], respectively, at the wavevector $\mathbf{k}_1$ and its main harmonics, and comparison of the field-induced variation of the harmonics intensities with that of $\mathbf{k}_1$. For each momentum transfer **Q** are given the magnetic wavevectors $n\mathbf{k}_1$, where $1 \leq n \leq 10$ is an integer, of the contributing harmonics and the corresponding structural Bragg peak τ, verifying $\mathbf{Q} = \tau + n\mathbf{k}_1$ or $\mathbf{Q} = \tau - n\mathbf{k}_1$.

Momentum transfer **Q**	Magnetic wavevector $n\mathbf{k}_1$	Structural Bragg position τ	Number of shots	$I_0[0;34T]$ (counts/s)	$I_m[35;39T]$ (counts/s)	$\frac{I_m(\mathbf{Q})-I_0(\mathbf{Q})}{I_m(\mathbf{Q}_1)-I_0(\mathbf{Q}_1)}$
(0.2 0 0)	$3\mathbf{k}_1$ = (1.8 0 0)<br>$7\mathbf{k}_1$ = (4.2 0 0)	(2 0 0)<br>(-4 0 0)	50	278 ± 6	259 ± 18	(-1.4 ± 1.4) %
(0.4 0 0)	$4\mathbf{k}_1$ = (2.4 0 0)<br>$6\mathbf{k}_1$ = (3.6 0 0)	(-2 0 0)<br>(4 0 0)	20	292 ± 10	268 ± 29	(-1.8 ± 2.2) %
$\mathbf{Q}_1$ = (0.6 0 0)	$\mathbf{k}_1$ = (0.6 0 0)<br>$9\mathbf{k}_1$ = (5.4 0 0)	(0 0 0)<br>(6 0 0)	54	256 ± 6	1627 ± 42	1
(0.8 0 0)	$2\mathbf{k}_1$ = (1.2 0 0)<br>$8\mathbf{k}_1$ = (4.8 0 0)	(2 0 0)<br>(-4 0 0)	20	272 ± 9	212 ± 25	(-4.4 ± 2.0) %
(1 0 0)	$5\mathbf{k}_1$ = (3 0 0)	(-2 0 0)	41	705 ± 11	604 ± 30	(-7.4 ± 2.4) %
(1 0 -1)	$0\mathbf{k}_1$ = (0 0 0)<br>$10\mathbf{k}_1$ = (6 0 0)	(1 0 -1)<br>(-5 0 -1)	58	19138 ± 46	19236 ± 156	(7.2 ± 11.9) %

Supplementary Note 1

Amplitude of the ordered magnetic moment

Assuming magnetic moments aligned along the easy magnetic axis **c**, the amplitude of the magnetic moment associated with a wavevector $\mathbf{k}_M$ is given by:

$$M(\mathbf{k}_M) = \frac{1}{f_M p|(\sin\alpha)|}\left|\sum_j b_j e^{i\mathbf{Q}_N\cdot\mathbf{r}_j}\right|\sqrt{\frac{I_M / L_M}{I_N / L_N}} \quad , \tag{1}$$

where $\mathbf{Q}_N$ is the momentum transfer at a nuclear Bragg position, I_M and I_N are the measured intensities (integrated over the sample precession angle ω) at $\mathbf{Q}_M$ (corresponding to the magnetic wavevector $\mathbf{k}_M$) and $\mathbf{Q}_N$, respectively, L_M and L_N are the Lorentz factors at $\mathbf{Q}_M$ and $\mathbf{Q}_N$, respectively, f_M is the magnetic form factor at $\mathbf{Q}_M$, $p = 0.2696.10^{-12}$ cm, α is the angle between the moment and **Q**, and b_j and $\mathbf{r}_j$ are the neutron scattering length and position of the ions j, with $b_{\mathrm{U}} = 0.842.10^{-12}$ cm, $b_{\mathrm{Ru}} = 0.721.10^{-12}$ cm, and $b_{\mathrm{Si}} = 0.4149.10^{-12}$ cm [1].

Here, we use Supplementary Equation (1), assuming a magnetic form factor corresponding to localized or nearly-localized f-electrons (estimated from a low-field study of the magnetic form factor [2]). From the intensity measured (and integrated over ω scans) at the magnetic Bragg peak at **Q** = (0.6 0 0) and at the nuclear Bragg peak at $\mathbf{Q}_N$ = (1 0 -1), we extract the moment amplitude $M(\mathbf{k}_1) = 0.24 \pm 0.02$ μ_B/U. From the field-induced magnetic Bragg peak at **Q** = (1.6 0 -1), which is equivalent to that measured at **Q** = (0.6 0 0) but whose intensity is roughly four-times smaller (cf. Figure 1), we extract a similar value $M(\mathbf{k}_1) = 0.27 \pm 0.03$ μ_B/U. We also estimate that, for $35 < \mu_0 H < 39$ T, an increase of $\simeq$ 2000 - 8000 counts/s would be expected at **Q** = (1 0 -1) if the magnetization of 0.5 - 1 μ_B/U was related to a magnetic Bragg peak at $\mathbf{k}_{ZC}$ = (0 0 0).

Supplementary Note 2

Spin density wave vs. squared modulation

Assuming a multi-**k** structure (made either of harmonics of the main wavevector or of equivalent wavevectors), the moment on the U site of position **r** is given by the superposition of the sine-modulated moments associated with each component $\mathbf{k}_i$:

$$M_U(\mathbf{r}) = \sum_{i=1}^{N} M_U(\mathbf{k}_i, \mathbf{r}) , \tag{2}$$

where N is the number of considered wavevectors,

$M_U(\mathbf{k}_i,\mathbf{r}) = M(\mathbf{k}_i)\cos(2\pi\mathbf{k}_i\mathbf{r} + \varphi_i)$ if $\mathbf{k}_i$ is at the Brillouin zone center,

$M_U(\mathbf{k}_i,\mathbf{r}) = 2M(\mathbf{k}_i)\cos(2\pi\mathbf{k}_i\mathbf{r} + \varphi_i)/N$ if $\mathbf{k}_i$ is at a Brillouin zone border shared by N zones,

and $M_U(\mathbf{k}_i,\mathbf{r}) = 2M(\mathbf{k}_i)\cos(2\pi\mathbf{k}_i\mathbf{r} + \varphi_i)$ for all other $\mathbf{k}_i$.

Supplementary Table 1 presents, for several momentum transfers **Q** corresponding to the harmonics $n\mathbf{k_1}$, the neutron intensities I_0 integrated in the field window [0;34T] in the hidden-order phase and I_m integrated in the field window [35;39T] in the field-induced ordered phase, resulting from summations over 20 to 58 pulsed fields shots. The ratios between [$I_m(\mathbf{Q})$ - $I_0(\mathbf{Q})$] and [$I_m(\mathbf{Q}_1)$ - $I_0(\mathbf{Q}_1)$], which are the field-induced variations of intensity at **Q** and $\mathbf{Q}_1$, respectively, indicate that no magnetic Bragg peak develops at the harmonics in high magnetic field, within an error of 5-10 % of the magnetic intensity at $\mathbf{k}_1$ (5 % error or less for **Q** = (0.2 0 0), (0.4 0 0), (0.8 0 0), and (1 0 0), and 12 % error for **Q** = (1 0 -1)). The non-observation of harmonics within these experimental errors permits to rule out the picture of a squared magnetic structure of URu_2Si_2 between 35 and 39 T and, thus, to support the picture of a spin-density wave with the wavevector $\mathbf{k}_1$ = (0.6 0 0).

Supplementary references

[1] Lovesey, S. W. Theory of neutron scattering from condensed matter, Volume 1: Nuclear Scattering (Clarendon Press, Oxford, 1984).

[2] Kuwahara, K., Kohgi, M., Iwasa, K., Nishi, M., Nakajima, K., Yokoyama, M., & Amitsuka, H. Magnetic form factor of URu_2Si_2. *Physica B* **378-380**, 581-582 (2006).
